# Supplementary material for: Chalcone-Acridine Hybrid Suppresses Melanoma Cell Progression via G2/M Cell Cycle Arrest, DNA Damage, Apoptosis, and Modulation of MAP Kinases Activity
Source: Int J Mol Sci. 2022 Oct 14;23(20):12266. doi: 10.3390/ijms232012266 (PMC9603750; doi:10.3390/ijms232012266)
Supplement: Supplementary file 1 [file ijms-23-12266-s001.zip › ijms-1931468-supplementary.pdf]

International Journal of
Molecular Sciences

MDPI

Supplement file

Chalcone-acridine hybrid suppresses melanoma cell progression via G2/M cell cycle arrest, DNA damage, apoptosis, and modulation of MAP kinases activity

Maria Gazdova [1], Radka Michalkova [1], Martin Kello [1], Maria Vilkova [2], Zuzana Kudlickova [2], Janette Baloghova [3], Ladislav Mirossay [1] and Jan Mojzis [1,*]

[1] Department of Pharmacology, Faculty of Medicine, Pavol Jozef Šafárik University, 040 01 Košice, Slovakia
[2] NMR Laboratory, Institute of Chemistry, Faculty of Science, Pavol Jozef Šafárik University, 040 01 Košice, Slovakia
[3] Department of Dermatovenerology, Faculty of Medicine, Pavol Jozef Šafárik University, 040 01 Košice, Slovakia
* Correspondence: jan.mojzis@upjs.sk

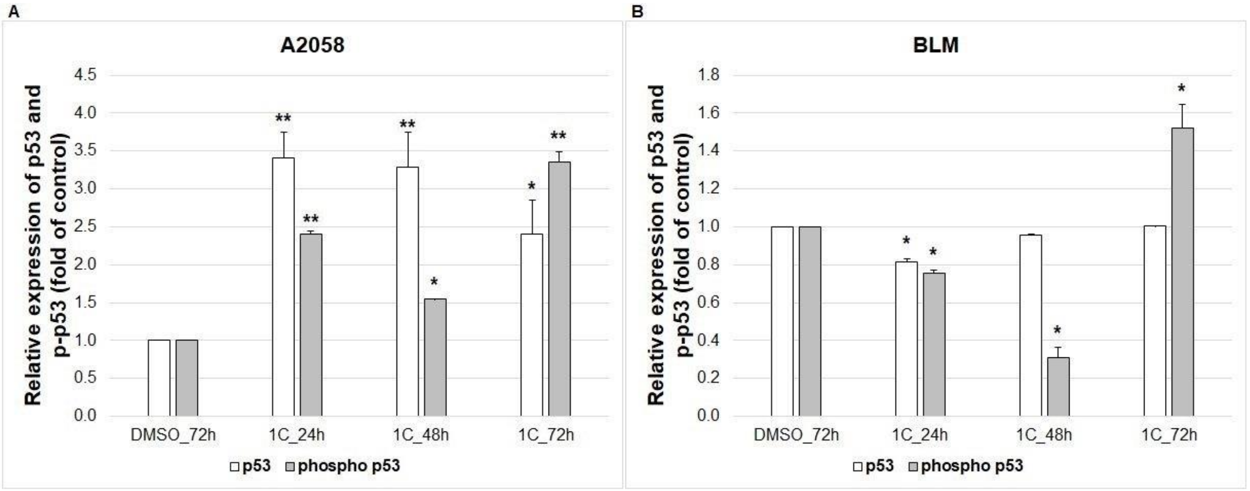

Figure S1 Densitometry analysis of total and phosphor p53 protein WB in (**A**) A2058 and (**B**) BLM melanoma cells. * $p < 0.05$, ** $p < 0.01$ DMSO.

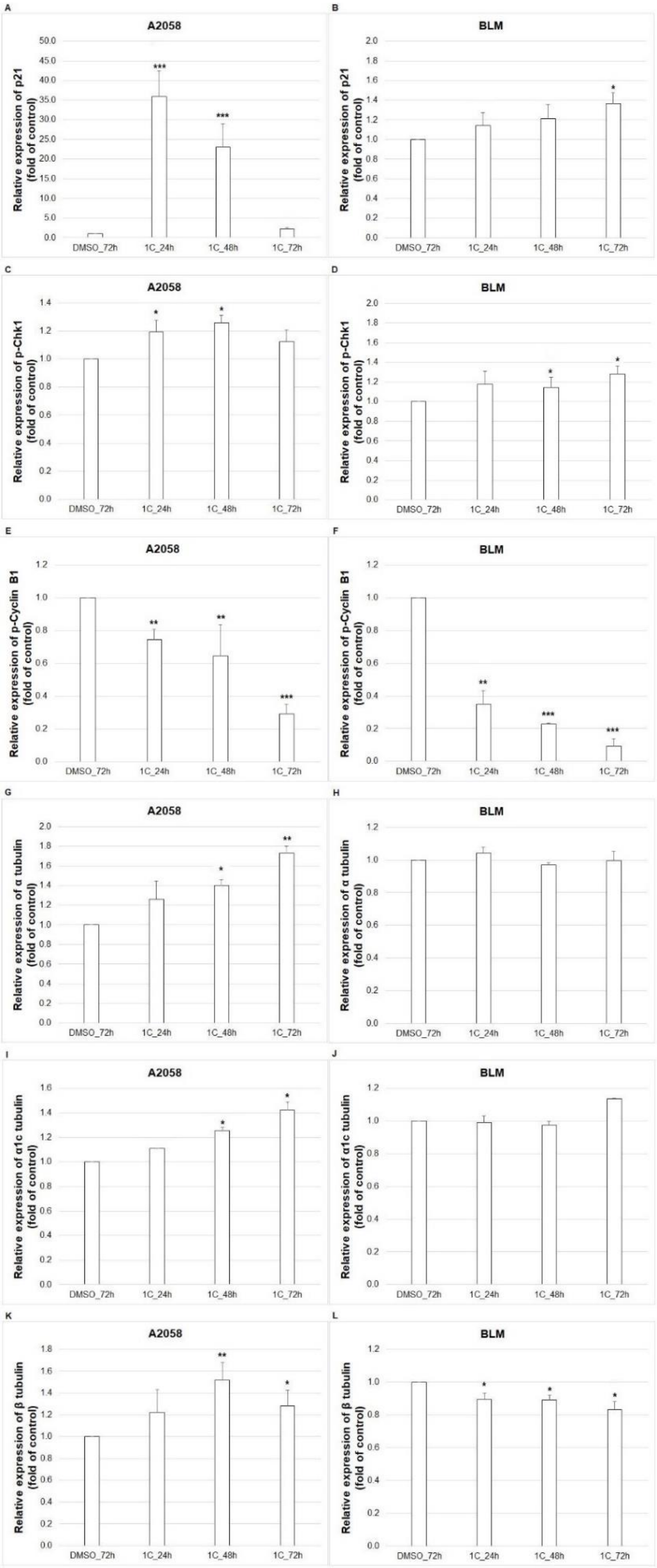

Figure S2 Densitometry analysis of p21, p-Chk1, p-Cyclin B1, α-/α1c-/β tubulins in (**A**,**C**,**E**,**G**,**I**,**K**) A2058 and (**B**,**D**,**F**,**H**,**J**,**L**) BLM melanoma cells. * $p < 0.05$, ** $p < 0.01$, *** $p < 0.001$ vs. DMSO.

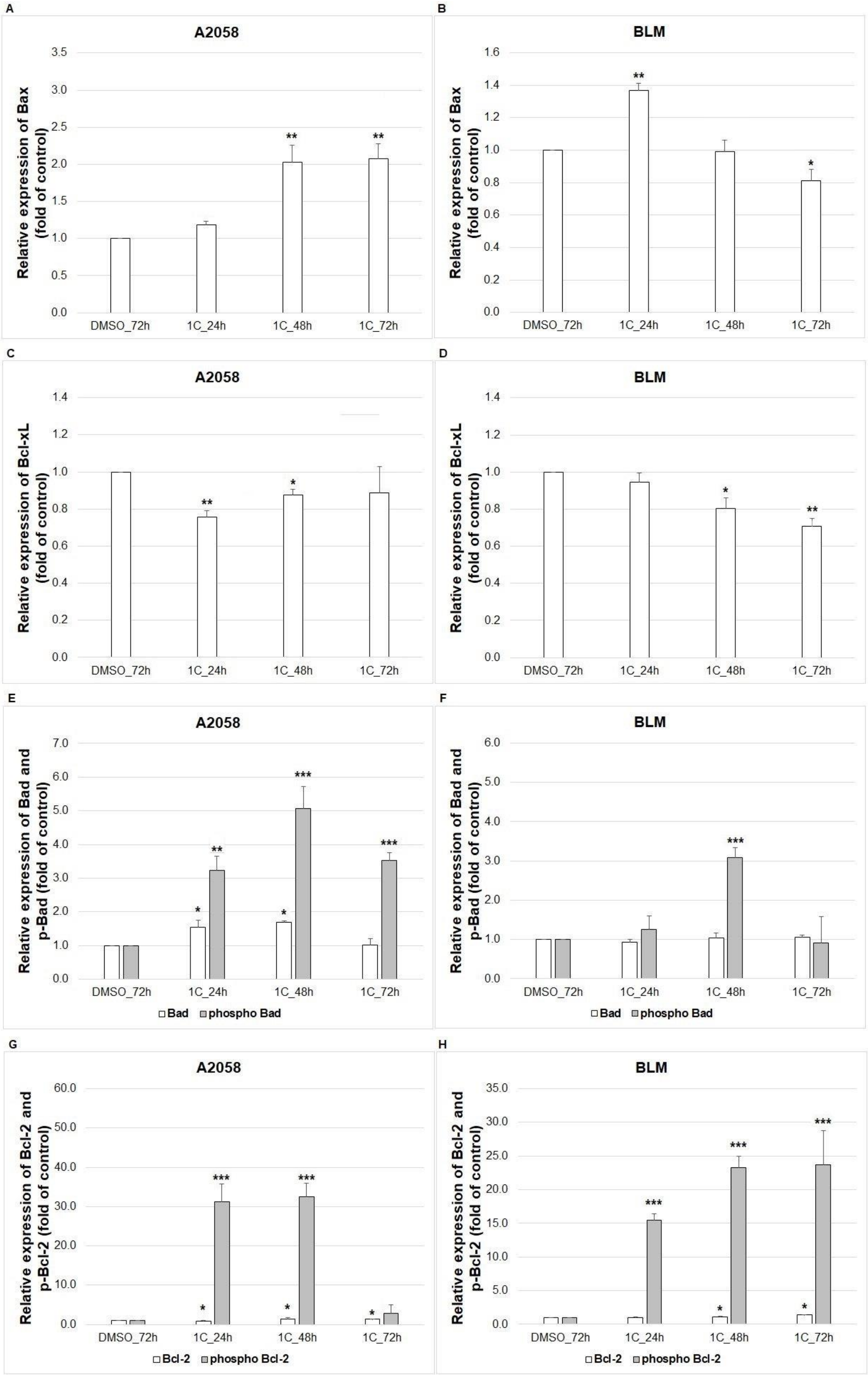

Figure S3 Densitometry analysis of Bax, Bcl-xL, Bad and Bcl-2 proteins in (**A**,**C**,**E**,**G**) A2058 and (**B**,**D**,**F**,**H**) BLM melanoma cells. * $p < 0.05$, ** $p < 0.01$, *** $p < 0.001$ vs. DMSO.

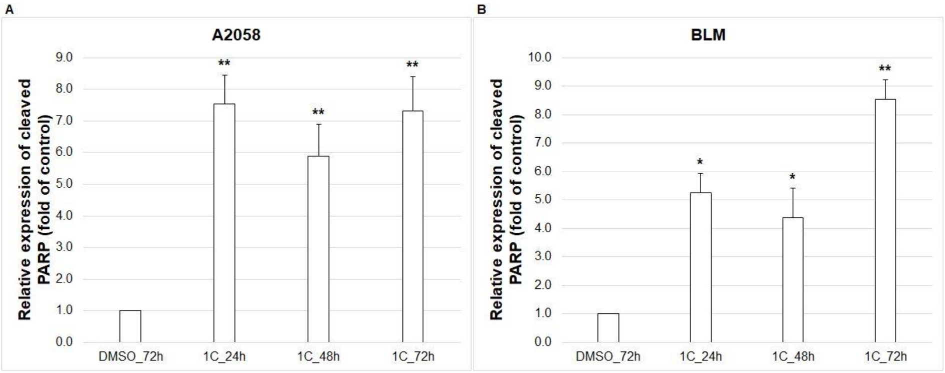

Figure S4 Densitometry analysis of PARP proteins cleavage in (**A**) A2058 and (**B**) BLM melanoma cells. * $p < 0.05$, ** $p < 0.01$ vs. DMSO.

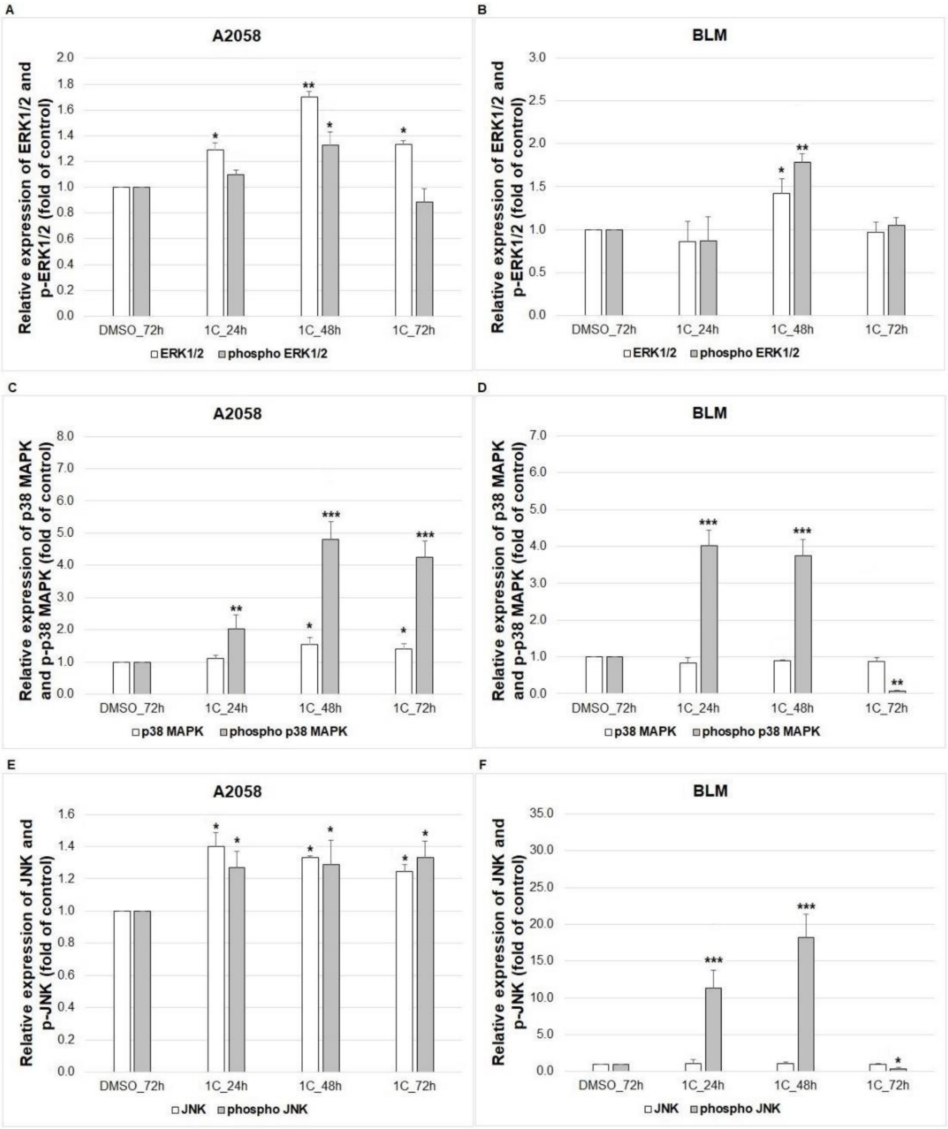

Figure S5 Densitometry analysis of ERK 1/2, p38 MAPK, JNK proteins in (**A**,**C**,**E**) A2058 and (**B**,**D**,**F**) BLM melanoma cells. Statistical significance: * $p < 0.05$, ** $p < 0.01$, *** $p < 0.001$ vs. DMSO.
